# Quantum Mechanical Molecular Interactions for Calculating the Excitation Energy in Molecular Environments: A First-Order Interacting Space Approach

**DOI:** 10.1002/cphc.201402635

**Published:** 2014-11-13

**Authors:** Jun-ya Hasegawa, Kazuma Yanai, Kazuya Ishimura

**Affiliations:** [a] Catalysis Research Center, Hokkaido University Kita 21, Nishi 10, Kita-ku, Sapporo, 011-0021 (Japan) E-mail: hasegawa@cat.hokudai.ac.jp; [b] JST-CREST 4-1-8 Honcho, Kawaguchi, Saitama, 332-0012 (Japan); [c] Department of Theoretical and Computational Molecular Science, Institute for Molecular Science 38 Nishigo-Naka, Myodaiji, Okazaki 444-8585 (Japan)

**Keywords:** decomposition analysis, dispersion effects, excited states, polarization effects, solute–solvent clusters

## Abstract

Intermolecular interactions regulate the molecular properties in proteins and solutions such as solvatochromic systems. Some of the interactions have to be described at an electronic-structure level. In this study, a commutator for calculating the excitation energy is used for deriving a first-order interacting space (FOIS) to describe the environmental response to solute excitation. The FOIS wave function for a solute-in-solvent cluster is solved by second-order perturbation theory. The contributions to the excitation energy are decomposed into each interaction and for each solvent.

The properties of solutes are often relevant to the interactions with environmental (solvent) molecules via steric repulsions, electrostatic interactions, orbital interactions, exchange repulsions, and dispersion interactions.[1] A typical example is solvatochromism,[2] in which the interactions modulate the relative energy levels of the excited states. Color tuning in photobiological systems (e.g. vision,[3] bioluminescence,[4] and engineered fluorescent proteins[5]) is the result of biological solvatochromism due to the protein environment. Another significant example of environmental energy tuning is photoinduced electron transfer (PIET).[6] In electron donor–acceptor systems, the electron-transfer rate depends on the solvent polarity. A pioneering theoretical study illustrated an essential role of the polarization effect in photosynthetic PIET using a continuum model.[7]

Theoretical methods and their applications to photobiological color tuning were comprehensively summarized in a recent review article.[8] There are several classes of multiscale and multiphysics approaches for embedded solute-in-solvent systems, such as continuum models,[9] hybrid quantum mechanical (QM)/molecular mechanical (MM) methods,[10] our-own *n*-layered integrated molecular orbital and molecular mechanics method (ONIOM),[11] effective fragment potential (EFP),[12] frozen density embedding (FDE),[13] and so on. Concerning the excited-state polarization effect in protein environments, the QM/MM method was extended to triple-layer QM/QM/MM,[14] in which the second QM region was described by density functional theory (DFT) and was coupled with the first QM region via electrostatic interactions.

We have also focused on molecular interactions in the excited states of photobiological systems.[15] Our QM/MM code was applied to clarify the electrostatic color-tuning mechanism in protonated retinal Schiff base,[16] firefly luciferase,[17] and fluorescent protein.[18] The symmetry-adapted cluster-configuration interaction (SAC-CI) method[19] was applied for the core QM region. To investigate the roles of the QM environmental effect, an ONIOM type triple-layered QM/QM/MM calculation was performed.[20] For the low-level QM method that describes a solute-in-solvent cluster, CI singles (CIS) was adopted to consider the orbital delocalization and excitonic coupling effects in calculated excited states. In bacteriorhdopsin20a, b and fluorescent protein,18b the second QM layer corrected the numerical results to the right directions.

At this point of the study, our question is how far we have to go up to higher-order excited-state molecular interactions to improve our understanding of the tuning mechanism. It is better to introduce a criterion for truncating the interaction hierarchy based on a right reason. The subject of the present study is, therefore: 1) to derive an operator that defines the excitation energy (presently at the Hartree–Fock (HF)/CIS level), 2) to derive a first-order interaction space (FOIS) of the operator to investigate the physical interpretation of the FOIS, 3) to propose a wave function and a scheme to solve the wave function to obtain the correction to the excitation energy, and 4) to decompose the correction into intermolecular interactions.

Here, we assume HF and CIS solutions for the ground and excited states of a solute-in-solvent cluster model, respectively [Eq. (1)]:


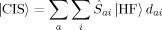
(1)


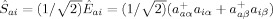
 is a normalized spin-symmetry adapted excitation operator. The CIS coefficients are represented by {*d_ai_*}. The HF MOs were assumed to be properly localized within one of the fragments. In Figure 1, the definitions of the MO indices are illustrated. {*i*,*j*⋅⋅⋅} and {*a*,*b*⋅⋅⋅} are occupied and virtual MOs of any unspecified fragments, respectively. MOs in the *n*th fragment are represented by {*i*^*n*^,*a*^*n*^}. Solute is defined as the 0th fragment.

**Figure 1:**
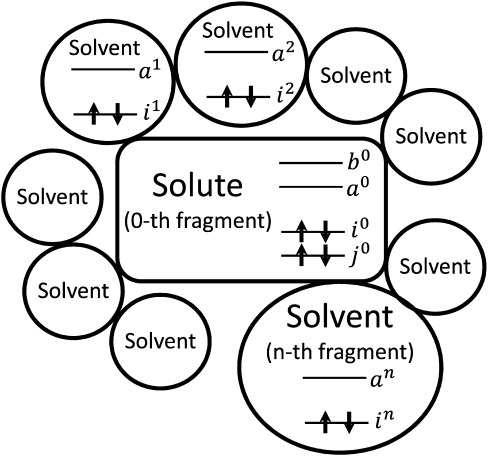
Definitions of the molecular orbital (MO) indices of a solute-in-solvent system.

We consider the space generated by 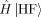 and 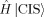 (see explicit formula in the Supporting Information, SI). 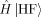 and 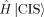 generate configurations up to double and triple excitations, respectively. This information includes not only the energy of the HF and CIS states but also the corrections defined by the FOIS.

Because our interest is in calculating the excitation energy, we derive a commutator as below [Eq. (2)]:


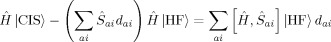
(2)

This kind of commutator was derived for calculating the excitation energy for the coupled-cluster ground-state wave function.[21] We rewrite this operator as 
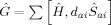
. 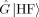 involves up to double excitations. There is no triple excitation because triples in 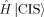 are completely cancelled by double excitations in 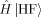 in Equation (2). 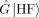 gives excitation energy at the HF/CIS level, 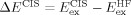, when 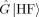 is projected on single-excitation manifold, such as 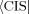. The double excitations in the 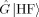 represent correction to the excitation energy, Δ*E*^CIS^.

Next we want to understand how single excitations at the solute (0th fragment) interact with the solvents’ excitations. For this purpose, localized molecular orbitals (LMOs) are introduced, and each MO is assigned to one of the fragments. A set of single excitations within the solute moiety is used for constructing the 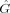 operator, 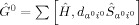. To obtain the FOIS, which is important for calculating the excitation energy, we take three steps: First, the 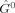 operator was applied to the HF state. Second, the excitations within the solute were projected out by using the projection operator 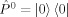, where 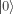 represents the excitations within the solute’s MO space. Finally, we neglected the terms involving two-electron repulsion integrals such as (*b^n^j*^*m*^|*i*^0^*a*^0^), *n*≠*m*. In our previous study, we found that those integrals were small enough not to affect the calculated energy20c because the product of LMO, *b^n^j*^*m*^, is very small, when the two belong to different fragments. These steps derive the effective FOIS for the 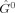 operator as follows [Eqs. (3)]:


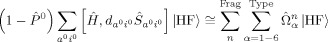
(3)

We introduce six types of operators 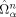
(*α*=1–6) to represent the component of the FOIS. A schematic diagram for the operators is shown in Figure 2. The first two operators [Eqs. (4) and (5)]:[Disp-formula m5]


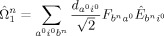
(4)


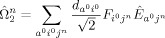
(5)

**Figure 2:**
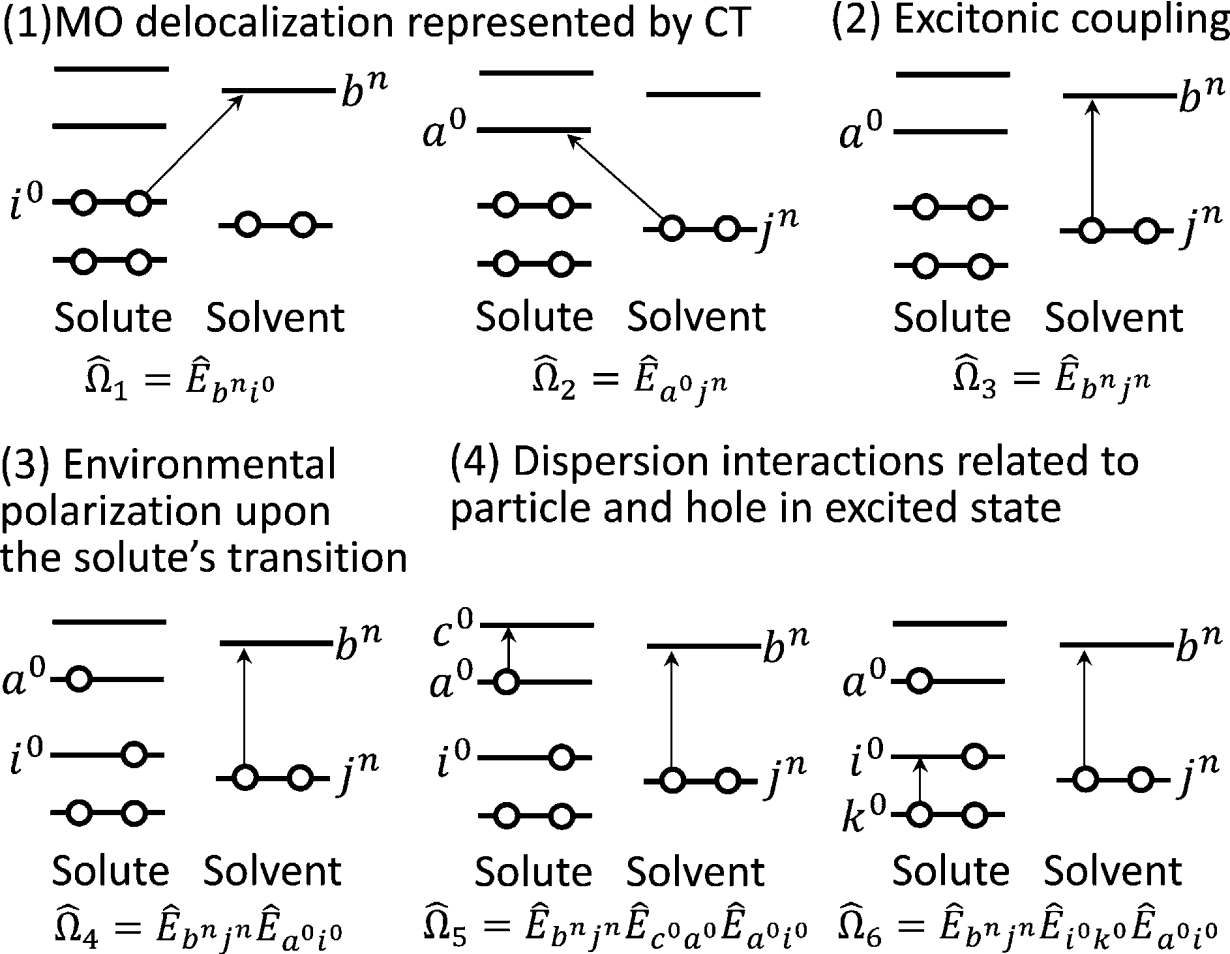
Six types of the FOIS operators 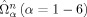.

express charge-transfer (CT) excitations from solute *i*^0^ to solvents *b*^*n*^ and those from solvent *j*^*n*^ to solute *a*^0^, respectively. These operators describe the delocalization effect of the MOs between solute and solvents. The Fock matrix elements represent the magnitudes of the orbital mixings.

The third one represents one-electron local excitations (LEs) within each solvent fragment [Eq. (6)]:


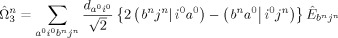
(6)

The integrals (*b^n^j*^*n*^|*i*^0^*a*^0^) and (*b^n^a*^0^|*i*^0^*j*^*n*^) describe the Coulomb and exchange interactions between the solute’s excitation *i*^0^→*a*^0^ and the solvents’ excitations *b*^*n*^→*j*^*n*^, respectively. These interactions can also be understood in terms of the excitation energy transfer theory. The former and latter terms correspond to Förster- and Dexter-type interactions between donor and acceptor, respectively.

The 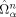
(*α*=1–3) operators are single excitations and have been already included in the CIS wave function of the solute-in-solvents cluster. The numerical importance of these operators has been investigated in our previous studies.[20]

The forth operators are double excitations that are composed of the solvents’ single excitations on top of the solute’s single excitations [Eq. (7)]:


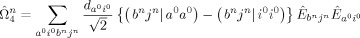
(7)

These operators represent the polarization of the solvents’ electronic structure upon solute excitation because the repulsion integrals that appear in Equation (7) are rewritten as [Eq. (8)]:


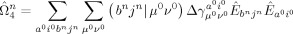
(8)

where *μ*^0^ and *ν*^0^ denote the atomic orbitals of the solute moiety; 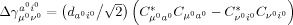 is the difference in electron density associated with the excitation from *i*^0^ to *a*^0^; 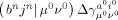 indicates the magnitude of solvent polarization due to the change in the solute’s charge distribution upon excitation.

The fifth and sixth operators represent dispersion interactions that are specific in the excited state [Eqs. (9) and (10)]: [Disp-formula m9], [Disp-formula m10]


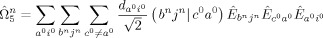
(9)


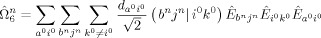
(10)

where 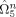 describes the double excitation, that is, the one-electron virtual-to-virtual excitations at the solute, *a*^0^→*c*^0^, that couples with another one-electron excitation at the solvent, *j*^*n*^→*b*^*n*^. 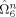 is also a double excitation. The one-electron excitation at the solute fills a hole in the excited state of the solute, and another one-electron excitation at the solvent represents the polarization of the solvent.

It is noteworthy that dispersion-type excitations, which are common to the ground and excited states, do not appear in the present formula. For instance, a double excitation such as 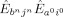, which is included in the FOIS of the 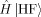 expansion (see SI), disappears. This is because the commutator (2) cancels the operators with a corresponding triple excitation operator in the excited state. This fact simplifies the components of the FOIS operators and significantly reduces the computational effort for evaluating the molecular interaction effect. A previous study on the dispersion effect in the excited states[22] also showed that important portions of the dispersion effects in the ground and excited states cancel each other in the HF and CIS model.

Next, a scheme for solving the wave function in the FOIS is explained. At our starting point, we assume that HF and CIS solutions for a solute-in-solvent system with LMO reference are at our disposal. This means that the effects of 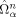
(*α*=1–3), which represent MO delocalizations and exciton couplings, are already taken into account at the CIS solution of the solute-in-solvent system. We therefore focus on solving the equations for the 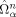
(*α*=4–6) operators. Because these operators act on the CIS state, a possible form of the wave function is [Eqs. (11)–(14)]: [Disp-formula m11], [Disp-formula m12], [Disp-formula m13], [Disp-formula m14]


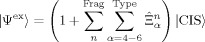
(11)


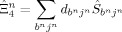
(12)


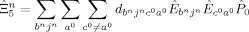
(13)


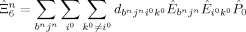
(14)

This form of the wave function is similar to that of the multi-configuration CI singles and doubles with an internally contracted FOIS. Each 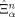 operator arises from the 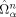 operator. One problem of the space generated by the three operators (12–14), is singularity. First, the excitations generated by 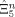 are singular—and those by 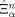 have the same problem. Second, the three operators are double excitations from the HF state, and therefore, they overlap with each other. This strong singularity causes not only a numerical instability but also ambiguity in the decomposition analysis. In the present study, we first evaluate the entire contribution from the 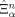
(*α*=4–6) operators, 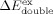, by adopting double excitations in the wave function as follows [Eq. (15)]:


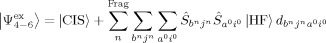
(15)

Here, the CIS state vector in the second term has been de-contracted. To evaluate the polarization effect, 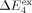, a wave function containing the 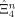 operator is solved [Eq. (16)]:


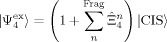
(16)

The dispersion contributions, 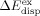, were evaluated as 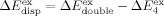. The wave functions in Equations (15) and (16) were solved by the second-order perturbation theory for each fragment. 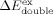 and 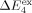 are defined as the sum of the fragments’ contributions, 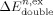 and 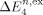, respectively. Details of the derivation are given in the SI.

Below we show the results of the pilot applications of the present method. We selected the *n*–π* and π–π* excited states of *s*-*trans*-acrolein (ACL) and the π–π* excited state of methylenecyclopropene (MCP) in water clusters (Figure 3). These states show large solvatochromic shifts and have been targets for theoretical calculations including solvation effects[23] (see the references cited in Ref. [24]).

**Figure 3:**
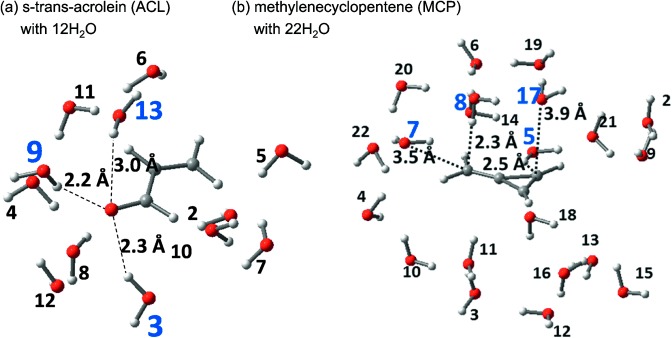
Computational models of: a) ACL with 12 H_2_O molecules and b) MCP with 22 H_2_O molecules. For the geometry, see the computational details. The indices in blue denote fragments with a relatively large contribution to the calculated excitation energy.

HF and CIS calculations were performed for the clusters. We applied our localization scheme[25] to the HF MOs and localized the MO distribution within each fragment (solute and solvent). Using this LMO basis, the CIS configurations are classified into groups of excitations, such as excitations within a fragment and CT between fragments. To visualize each component in the CIS wave function, we adopted a plot, max |*d_ai_*| plot (see Figure 4), which was used in our previous studies.20b, c The contribution from each excitation group was represented by the maximum value within the group, {|*d_ai_*|}.

**Figure 4:**
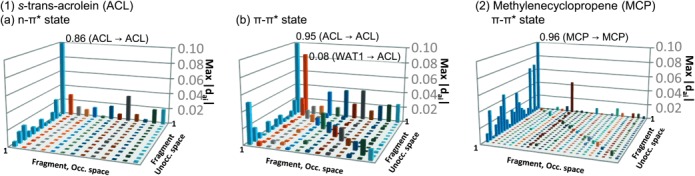
Max|*d_ai_*| plot of: 1a) the *n*–π* state and 1b) the π–π* state of ACL in the water cluster; and 2) the π–π* state of MCP in the water cluster. ACL and MCP are fragment 1 in the figures. The blue bars in the leftmost line correspond to excitations from fragment 1 (solute) to other fragments; the bars in the far side correspond to excitations to fragment 1; and the bars in the diagonal line are local excitations in a fragment. “WAT1” denotes one of the water molecules.

First, we explain the ACL results. The calculated excitation energies are summarized in Table 1. For the *n*–π* state, the calculated excitation energy with the TIP3P point-charge model (CIS:TIP3P) is 4.91 eV. As the CIS description expanded to the whole water cluster, the excitation energy increased by 0.07 eV and became 4.98 eV. There are two reasons to explain the shift. The first one is that the description of the electrostatic interactions is improved and the exchange interaction is introduced. The second one is that orbital delocalization and excitonic interactions are included. In this case, the shift can be explained by the former reason because the latter effects usually decrease the excitation energy in weakly interacting systems.

**Table 1:** Excitation energies of ACL and MCP in a water cluster calculated with several models for the environment, TIP3P (a point-charge model), CIS, and the present PT2 correction (units are in eV)

Model^[a]^	ACL^[b]^		MCP^[c]^
	*n*–π^*^	π–π^*^	π–π^*^
CIS:TIP3P	4.91	7.14	6.05
CIS:CIS	4.98	7.02	6.00
CIS:CIS+PT2	4.92	6.97	5.95
Δ^CIS+PT2^	+0.01	−0.17	−0.10

[a] “X:Y” denotes computational models for the “solute:solvent” system. Δ^CIS+PT2^ denotes an energy correction at the CIS+PT2 level. [b] The cc-pVTZ and cc-pVDZ basis sets were used for ACL and water molecules, respectively. [c] The cc-pVDZ sets was used for both MCP and water molecules.

For the π–π* state, the calculated excitation energies in the CIS:TIP3P and CIS:CIS models were 7.14 and 7.02 eV, respectively. Compared with the *n*–π* states, a different trend in the energy shift was observed in changing the solvent model. The decrease is due to both delocalization and excitonic contributions from the solvents. As seen in Figure 4 b, the result of the max|*d_ai_*| plot clearly shows the LE and CT contributions in the CIS wave function. The magnitude of the CIS coefficients is larger than that of the *n*–π* state. The difference is particularly large in the LE contributions that are related to the magnitude of the excitonic coupling between the solute excitations to the solvent ones. The excitonic coupling is well-approximated by the multipole expansion[26] that is proportional to the product of the transition dipole moment (TDM) between two excitons. The calculated TDM of the π–π* state (4.05 a.u.) is larger than that of the *n*–π* state (0.00 a.u.) at the CIS:TIP3P level.

The results for MCP are also summarized in Table 1. The calculated excitation energies at the CIS:TIP3P and CIS:CIS levels were 6.05 and 6.00 eV, respectively. The amount of decrease by the CIS description for the water cluster is 0.05 eV, which is less than the half of that in the π–π* state of ACL (0.12 eV). As seen in Figures 4–2, the contributions from LE are much smaller than those in the ACL. The reason is ascribed to the TDM of the π–π* state of MCP (0.48 a.u. at the CIS:TIP3P level), which is only 10 % of that of ACL.

Next, the results of the second-order perturbation correction to the excitation energy are explained. As described above, polarization and dispersion interactions in the excited states are included in this model. In the case of ACL, the total energy shifts due to the second-order contribution were −0.064 and −0.054 eV for the *n*–π* and π–π* states, respectively. As summarized in Table 1, the calculated excitation energy (CIS:CIS+PT2) of the two states became 4.92 and 6.97 eV, respectively. Consequently, the TIP3P point-charge description for the *n*–π* state gave an excitation energy very close to the CIS+PT2 one; the difference Δ^CIS+PT2^ is only +0.01 eV. On the other hand, both the CIS and PT2 corrections decreased the excitation energy of the π–π* state. The difference Δ^CIS+PT2^ became −0.17 eV. The Δ^CIS+PT2^ is regarded as our QM correction to the TIP3P excitation energy and will be used for the ONIOM type correction to the SAC-CI:TIP3P result.

In the case of the π–π* state of MCP, the second-order correction was calculated to be −0.050 eV, and the CIS:CIS+PT2 excitation energy was 5.95 eV. Similar to the π–π* state of ACL, both the CIS and PT2 corrections decreased the excitation energy, and the change from the TIP3P description was −0.10 eV.

A decomposition analysis was performed for the result of the second-order perturbation correction to the excitation energy. The polarization and dispersion contributions from the solvent are given in Figures 5 and 6, In the case of ACL, the dispersion contribution is the dominant contribution, 77 % and 93 % in the *n*–π* and π–π* states, respectively. In the π–π* state of MCL, the polarization effect shows a different feature. Interestingly, the polarization contribution was −0.026 eV and increased to 50 % in the second-order effect. This contrasts to those of the *n*–π* (−0.015 eV) and π–π* (−0.004 eV) states of ACL. It is possible to ascribe the origin of this trend to the difference of the dipole moments (DM) between the ground and excited states, |Δμ|. The DMs of the three states are summarized in Figure S5. For ACL, the DMs of the *S*_0_, *n*–π*, and π–π* states are 3.15, 1.04, and 3.56 Debye, respectively. For MCP, the DMs in the *S*_0_ and π–π* states are 2.06 and 4.47 Debye, respectively. Therefore, the |Δμ| value of the *n*–π* and π–π* states of ACL were 2.23 and 0.58 Debye, respectively, and that of the π–π* state of MCP were 6.53 Debye. The MO distributions qualitatively interpret the changes in the DM values. The trend in the polarization contributions correlates with the |Δμ| value very well.

**Figure 5:**
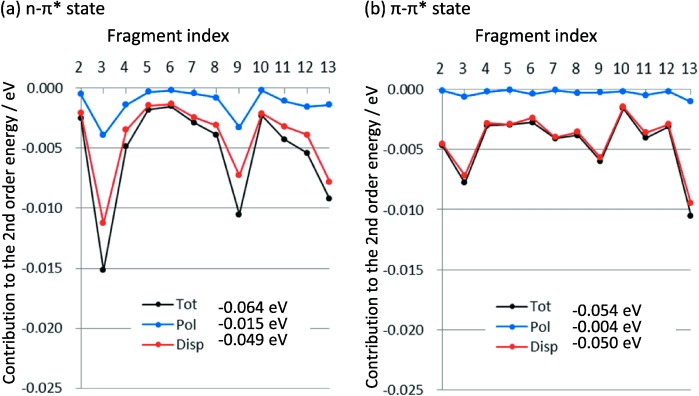
Calculated second-order contributions to the excitation energy of ACL with 12 H_2_O molecules: a) *n*–π* and b) π–π* states. See Figure 1 for the fragment indices.

**Figure 6:**
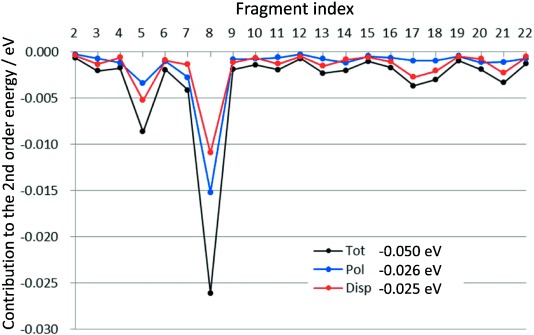
Calculated second-order contributions to the lowest π–π* excitation energy of MCP with 22 H_2_O molecules.

Figures 5 and 6 show the magnitude of the second-order energy contributions from each fragment. The solvents at closer distances to the solute tend to give larger contributions. For example, the third, ninth, and 13th fragments in the ACL water cluster gave relatively large contributions. These three are close to the O atom of ACL. Similarly in the MCP water cluster, the 5th and 8th solvents, which gave relatively large contributions, are at the close distance to MCP.

Before closing this paper, we compare the present result to the experimental data. We note, however, that there are two difficulties. The first one is that the present pilot applications adopted a cluster model to mimic the solution environment. The latter one is that the structure of the cluster model is just a snapshot of a classical trajectory. The comparison below is limited to a qualitative level. To estimate the excitation energies that are comparable to the experiment, we adopted the ONIOM scheme for including the electron correlation effect of the solute together with the QM corrections from the environment:[Disp-formula m17]


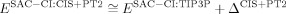
(17)

The excitation energies in the gas phase and the water cluster are shown in Table 2. The numbers in parenthesis represent the differences from the excitation energy in the gas phase.

**Table 2:** Calculated and experimental excitation energies of ACL and MCP in the gas phase and the water cluster. The numbers in parenthesis are the relative values of the excitation energy in the gas phase (units are in eV)

Model^[a]^	ACL		MCP
	*n*–π^*^	π–π^*^	π-π^*^
(1) in the gas phase			
SAC-CI^[b]^	3.57(0.00)	6.94(0.00)	4.81(0.00)
exptl.	3.75,^[c]^ 3.69^[d]^	6.41,^[f]^ 6.42^[d]^	
SAC-CI/aug-cc-pVDZ	3.83^[h]^	6.75^[h]^	4.47^[h]^
other theoretical	3.78,^[d]^ 3.85^[i]^	6.41,^[d]^ 7.15^[i]^	3.91^[j]^
(2) in the water cluster			
SAC-CI:TIP3P^[b]^	3.80(+0.23)	6.84(−0.10)	4.81(+0.00)
SAC-CI:CIS+PT2^[b]^	3.81(+0.24)	6.67(−0.27)	4.71(−0.10)
exptl.	3.94^[d]^(+0.2,^[h]^+0.25^[d]^)	5.89^[d]^(−0.4,^[h]^−0.53^[d]^)	4.49^[e]^
SAC-CI/aug-cc-pVDZ w/PCM	3.94(+0.11)^[h]^	6.61(−0.14)^[h]^	4.60(+0.13)^[h]^
other theoretical	4.04(+0.26)^[d]^ 4.09(+0.24)^[i]^	5.95(−0.46)^[d]^ 6.75(−0.40)^[i]^	4.45(+0.54)^[j]^

[a] Δ^CIS+PT2^ denotes the QM correction at the CIS+PT2 level (see Table 1). [b] For ACL and 12 H_2_O molecules, the cc-pVTZ and cc-pVDZ basis sets were used, respectively. For both MCP and 22 H_2_O molecules, the cc-pVDZ was used. [c] Ref. [27] [d] Result of “CAM-B3LYP MD QM/MM(SPC_pol_)+12 (H_2_O)_QM_”, Ref. [24a] [e] Ref. [28] [f] Ref. [29] [g] Ref. [30] [h] SAC-CI/aug-cc-pVDZ result, Ref. [31] [i] MRCISD+Q COSMO, Ref. [32] [j] For the gas and aqueous phases, the results for the *n*-pentane and methanol solutions are given. M06 w/IBSF protocol, Ref. [24 b].

For the *n*–π* state of ACL, the calculated results reasonably agree with the experimental ones. In the gas phase, the calculated excitation energy was 3.57 eV at the SAC-CI/cc-pVDZ level, while the experimental values were reported to be 3.75[27] and 3.69 eV.24a In the water cluster, the calculated excitation energy was 3.81 eV. The amount of the shift from the gas phase to the water cluster was calculated to be +0.24 eV, which is in good agreement with the experimental blue shift (+0.2[31] and +0.25 eV24a).

On the other hand, some systematic errors were observed for the π–π* states of ACL and MCP. The present SAC-CI calculation in the gas phase gave 6.94 eV for ACL, while the experimental values are 6.41[29]–6.4224a eV. This discrepancy results from the lack of basis sets and correlations described by connected triples. As shown in Table 2, a previous SAC-CI study showed that additional diffuse functions improved the result of ACL to be 6.75 eV.[31] The results were further improved to 6.65 eV by the CC3 wave function.24a In the calculations for the water cluster, we use the cc-pVDZ basis sets because of the tractable limit in our present code. In the case of MCP, no experimental data is available for the aqueous solution but for *n*-pentane one (4.01 eV[28]), which also suggests a similar discrepancy with the limitation of the computational method.

In the water cluster, the calculated excitation energies for the π–π* states of ACL and MCP were 6.67 and 4.71 eV, respectively, which overestimate the experimental ones as in the gas-phase calculations. These errors are also ascribed to the same origin as in the gas-phase situation. The experimental solvathochromic shifts for ACL (−0.4[31]–−0.5324a eV) were qualitatively reproduced by the present calculation (−0.27 eV). The discrepancy in the results could be reduced by using a more accurate wave function and by taking the MD sampling to reduce statistical errors.

In the present second-order evaluations, the polarization and dispersion effects on the excitation energy were collectively about −0.05 eV in the water cluster model, which are not an essential part of the solvatochromic effect. The magnitude of the polarization effect, however, depends on the change in the DM upon the transition. The significance of the polarization effect in the explicit charge-transfer systems is under investigation.

## Computational Details

The structures of ACL and MCP in the gas phase were optimized at the B3LYP/6-31G* level. The structures of the water clusters were determined with classical molecular dynamics trajectories (see details in the SI). The cluster models include solvents that have at least one of the atoms within 3 and 4 Å from ACL and MCP, respectively. The geometry of the ACL and MCP in the water clusters was further optimized at the B3LYP/cc-pVDZ and cam-B3LYP/cc-pVDZ levels, respectively. In the optimization, the water molecules were fixed at the MD structure and replaced by point-charges (TIP3P charges). We also fixed the C atom next to the O atom of ACL. In MCP, the atomic coordinates of the central C atom were fixed.

The HF orbitals were transformed into MOs localized within each fragment (solute and solvent). Our transformation[25] uses reference orbitals (RMOs) obtained with external calculations for isolated molecules. Overlap integrals between the RMOs and the transformed orbitals were maximized. In the SI, we show the populations at the fragments.

In the perturbation-selection step of the SAC-CI calculations, a set of threshold, “LevelFour”, was used.

All of the geometry optimizations were performed with the Gaussian 09 program.[33] For the excited states, our program was interfaced to the Gaussian program.

## Supporting Information

As a service to our authors and readers, this journal provides supporting information supplied by the authors. Such materials are peer reviewed and may be re-organized for online delivery, but are not copy-edited or typeset. Technical support issues arising from supporting information (other than missing files) should be addressed to the authors.

miscellaneous_information
