# Supplementary material for: Quantum Mechanical Molecular Interactions for Calculating the Excitation Energy in Molecular Environments: A First-Order Interacting Space Approach
Source: Chemphyschem. 2014 Nov 13;16(2):305–11. doi: 10.1002/cphc.201402635 (PMC4501320; doi:10.1002/cphc.201402635)
Supplement: Supplementary file 1 — miscellaneous_information [file cphc0016-0305-sd1.pdf]

CHEMPHYSCHEM

Supporting Information

© Copyright Wiley-VCH Verlag GmbH & Co. KGaA, 69451 Weinheim, 2015

Quantum Mechanical Molecular Interactions for Calculating the Excitation Energy in Molecular Environments: A First-Order Interacting Space Approach

Jun-ya Hasegawa,*[a, b] Kazuma Yanai,[a] and Kazuya Ishimura[c]

cphc_201402635_sm_miscellaneous_information.pdf

Supporting Information

Quantum Mechanical Effect of Molecular Environment: First-Order Corrections to Excitation Energy

Jun-ya Hasegawa,[a,b]* Kazuma Yanai,[a] and Kazuya Ishimura[c]

[a]Catalysis Research Center, Hokkaido University

Kita 21, Nishi 10, Kita-ku, Sapporo 001-0021, Japan

Tel & Fax: +81-11-706-9145

[b]JST-CREST

[c] Department of Theoretical and Computational Molecular Science

Institute for Molecular Science

38 Nishigo-Naka, Myodaiji, Okazaki 444-8585, Japan

* Corresponding author: E-mail: hasegawa@cat.hokudai.ac.jp

Contents

S-1. Explicit formula of the first-order interacting space (FOIS)

S-2. Derivation of the perturbation equations for solving the wave function

S-3. MO localization to predefined fragments: Löwdin population analysis

S-4. Complete list of authors in the reference 34 cited in the main text

S-5. MO distributions and dipole moment in the ground and excited states of ACL and MCP

S-6. Classical molecular dynamics calculations

S-7. Tables of the 2nd order contribution to excitation energy

S-8. Atomic coordinates of the computational models

S-1. Explicit formula of the first-order interacting space (FOIS)

Below is the explicit formula of the FOIS generated by multiplying the Hamiltonian operator to Hartree-Fock (HF) and configuration interaction singles (CIS) wave functions. MO indices, a, b, …, and i, j, …., indicate unoccupied and occupied orbitals, respectively. $\hat{E}_{ai}$ is a spin-adapted excitation operators that excites an electron in an occupied orbital *i* into an unoccupied orbital *a*. h_{pq} and F_{pq} denote a matrix element of one-electron and Fock operators, respectively. d_{ai} is a CIS wave function coefficient.

$$\hat{H}|\mathrm{HF}\rangle = \sum_{i}(h_{ii} + F_{ii})\,|\mathrm{HF}\rangle + \frac{1}{2}\sum_{bjck}(bj|ck)\hat{E}_{bj}\hat{E}_{ck}\,|\mathrm{HF}\rangle$$

$$= E_{\mathrm{g}}^{\mathrm{HF}}|\mathrm{HF}\rangle + \frac{1}{2}\sum_{bjck}(bj|ck)\hat{E}_{bj}\hat{E}_{ck}\,|\mathrm{HF}\rangle \tag{S1}$$

$$\hat{H}|\mathrm{CIS}\rangle = \sum_{ai}\left[\sum_{b}F_{ba}\hat{E}_{bi}\,|0\rangle - \sum_{j}F_{ij}\hat{E}_{aj}\,|0\rangle + \sum_{j}\{2(bj|ia) - (ba|ij)\}\hat{E}_{bj}\,|0\rangle\right]\frac{d_{ai}}{\sqrt{2}}$$

$$+\sum_{ai}\left[\sum_{bcj}(bj|ca)\hat{E}_{bj}\hat{E}_{ci}|0\rangle + \sum_{bjk}(bj|ik)\hat{E}_{bj}\hat{E}_{ak}|0\rangle + \frac{1}{2}\sum_{bjck}(bj|ck)\hat{E}_{ai}\hat{E}_{bj}\hat{E}_{ck}\,|0\rangle\right]\frac{d_{ai}}{\sqrt{2}}$$

$$= E_{\mathrm{ex}}^{\mathrm{CIS}}|\mathrm{CIS}\rangle$$

$$+\sum_{ai}\left[\sum_{bcj}(bj|ca)\hat{E}_{bj}\hat{E}_{ci}|0\rangle + \sum_{bjk}(bj|ik)\hat{E}_{bj}\hat{E}_{ak}|0\rangle + \frac{1}{2}\sum_{bjck}(bj|ck)\hat{E}_{ai}\hat{E}_{bj}\hat{E}_{ck}\,|0\rangle\right]\frac{d_{ai}}{\sqrt{2}} \tag{S2}$$

S-2. Derivation of the perturbation equations for solving the wave function

To determine the coefficients in eqs. (15) and (16), the wave function was inserted into Schrödinger equation. In the case of the wave function (15), the equation may be

$$\hat{H}|\mathrm{CIS}\rangle + \hat{H}\sum_{n}^{\mathrm{Frag}}\sum_{b^n j^n}\sum_{a^0 i^0}\hat{S}_{b^n j^n}\hat{S}_{a^0 i^0}|\mathrm{HF}\rangle d_{b^n j^n a^0 i^0}$$

$$= \Delta E_{double}^{ex}|\mathrm{CIS}\rangle + \Delta E_{double}^{ex}\sum_{n}^{\mathrm{Frag}}\sum_{b^n j^n}\sum_{a^0 i^0}\hat{S}_{b^n j^n}\hat{S}_{a^0 i^0}|\mathrm{HF}\rangle d_{b^n j^n a^0 i^0} \tag{S3}$$

and solved by using perturbation theory. $E_{ex}^{\mathrm{CIS+}}$ is the energy of the CIS state with the FOIS correction. Summing up the zero-th and first-order equations, we have

$$\langle\mathrm{CIS}|\left(\hat{H} - \Delta E_{double}^{ex(0-1)}\right)|\mathrm{CIS}\rangle = 0 \tag{S4}$$

$\Delta E_{double}^{ex(0-1)}$ denotes the sum of zeroth energy $\Delta E_{double}^{ex(0)}$ and first order energy $\Delta E_{double}^{ex(1)}$ and is equal to the CIS energy. The first-order wave function coefficients were determined by

$$\left\langle\Phi_{I^0 J^n}^{A^0 B^n}\right|\left(\hat{F} - \Delta E_{double}^{ex(0)}\right)\sum_{b^n j^n}\sum_{a^0 i^0}\hat{S}_{b^n j^n}\hat{S}_{a^0 i^0}|\mathrm{HF}\rangle d_{b^n j^n a^0 i^0} = -\left\langle\Phi_{I^0 J^n}^{A^0 B^n}\right|\hat{H}|\mathrm{CIS}\rangle \tag{S5}$$

where $\left\langle\Phi_{I^0 J^n}^{A^0 B^n}\right| = \langle\mathrm{HF}|\hat{S}_{I^0 A^0}^{\dagger}\hat{S}_{J^n B^n}^{\dagger}$. Because LMOs were used for the one-electron basis, Fock matrix involves non zero elements, and the eq. S4 were solved iteratively. The second-order energy is a summation of the energies of the fragments.

$$\Delta E_{double}^{ex(2)} = \sum_{n}^{\mathrm{Frag}}\Delta E_{double}^{n,ex(2)} \tag{S6}$$

$$\Delta E_{double}^{n,ex(2)} = \sum_{b^n j^n}\sum_{a^0 i^0}\langle\mathrm{CIS}|\hat{H}\hat{S}_{b^n j^n}\hat{S}_{a^0 i^0}|\mathrm{HF}\rangle d_{b^n j^n a^0 i^0} \tag{S7}$$

The equations for solving the $\hat{\Xi}_4^n$ operators were solved in the same way as equations S3-S7. The second-order energy is given by

$$\Delta E_4^{ex(2)} = \sum_{n}^{\mathrm{Frag}}\Delta E_4^{n,ex(2)} \tag{S8}$$

$$\Delta E_4^{n,ex(2)} = \sum_{b^n j^n}\langle\mathrm{CIS}|\hat{H}\hat{S}_{b^n j^n}|\mathrm{CIS}\rangle d_{b^n j^n} \tag{S9}$$

The dispersion effect was estimated as

$$\Delta E_{disp}^{ex(2)} = \sum_{n}^{\text{Frag}} \Delta E_{disp}^{n,ex(2)} = \sum_{n}^{\text{Frag}} \left(\Delta E_{double}^{n,ex(2)} - \Delta E_{4}^{n,ex(2)} \right) \tag{S10}$$

The polarization and dispersion contributions from each solvent were obtained from eq.s S9 and S10, respectively.

S-3. Fragment population based on the Löwdin population analysis

To determine localized molecular orbitals (LMOs), we used a localization procedure that we developed in our previous study.[1] A set of reference MOs (RMOs) was introduced, and the canonical MOs of total solute-solvent system was transformed by a unitary matrix that maximized overlap integrals with the RMOs. We used predefined RMOs that were calculated for each fragment as an isolated molecule.

The transformed MOs are sufficiently localized within each fragment. Figures S1 and S2 show fragment population for each MO in the acrolein + $12H_2O$ and methylenecyclopropene + $21H_2O$ systems, respectively. For instance, in Figure S1, MOs 1 to 16 are 1s core orbitals that are already local in their fragment. Each core MO has near 1.0 population in one of the fragments. MOs 17 to 27 are valence MOs of acrolein. These orbitals were transformed to be very close to their RMO by the localization procedure. As a result, these MOs populate on fragment 1 (acrolein). Not only occupied space but also unoccupied space was successfully transformed to LMOs localized within one of the fragments.

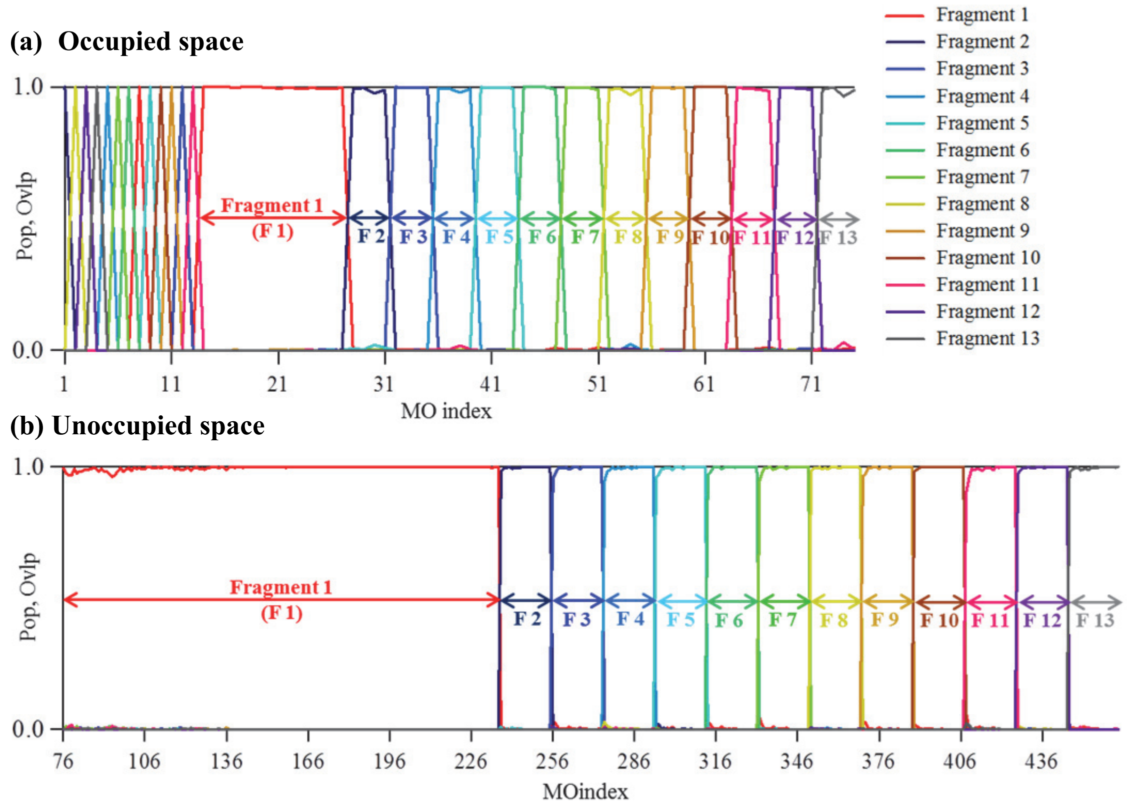

Figure S1. Fragment population analysis for s-trans-acrolein in water molecules. Löwdin analysis was performed to assign MO into one of the fragments. Results for (a) occupied MOs and (b) unoccupied MOs.

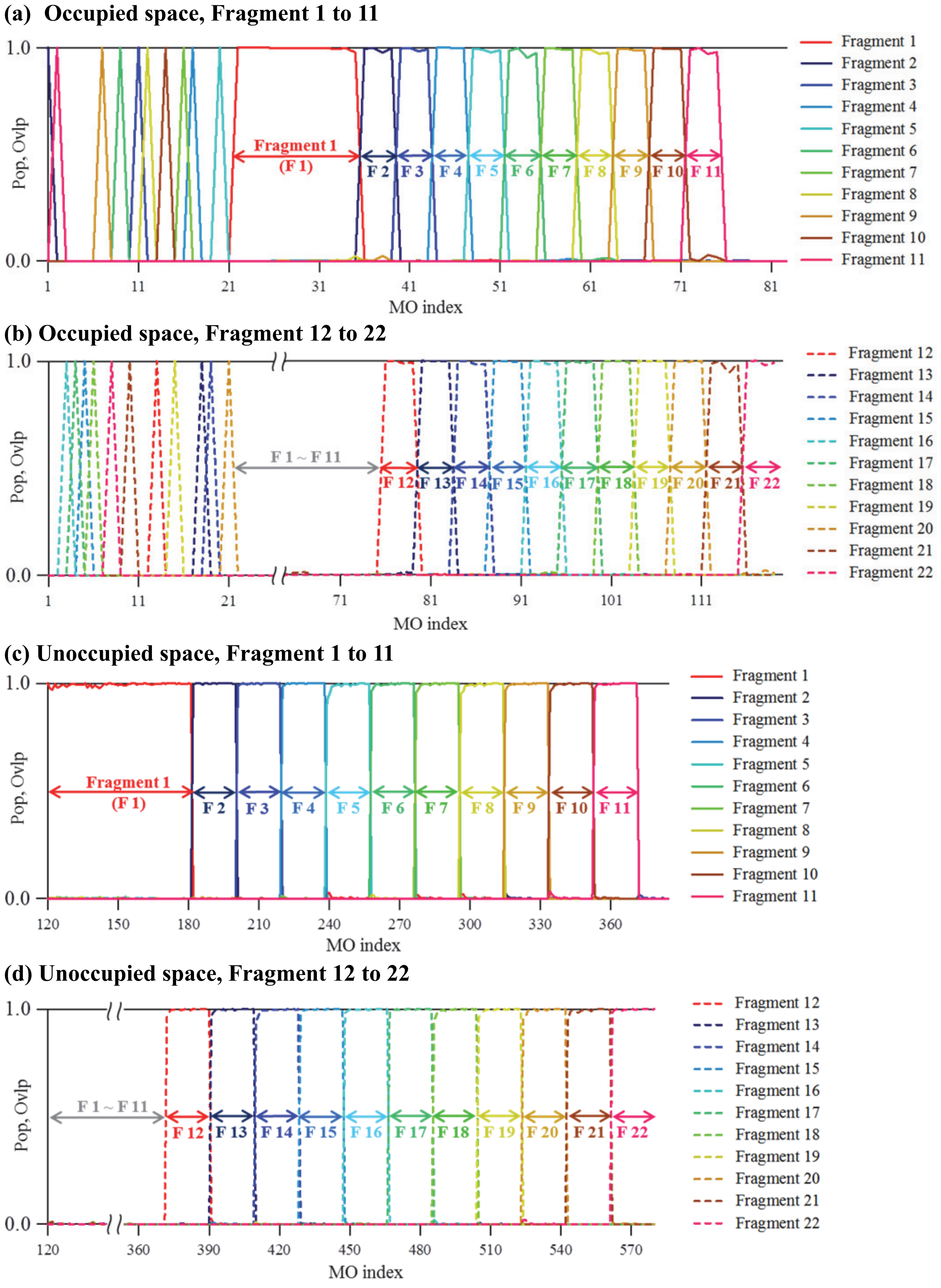

Figure S2. Fragment population analysis for methylenecyclopropene in water molecules. Löwdin analysis was performed to assign MO into one of the fragments. Results for (a and b) occupied space and (c and d) unoccupied space.

S-4. Complete list of authors in the reference 34 cited in the main text

M. J. Frisch, G. W. Trucks, H. B. Schlegel, G. E. Scuseria, M. A. Robb, J. R. Cheeseman, G. Scalmani, V. Barone, B. Mennucci, G. A. Petersson, H. Nakatsuji, M. Caricato, X. Li, H. P. Hratchian, A. F. Izmaylov, J. Bloino, G. Zheng, J. L. Sonnenberg, M. Hada, M. Ehara, K. Toyota, R. Fukuda, J. Hasegawa, M. Ishida, T. Nakajima, Y. Honda, O. Kitao, H. Nakai, T. Vreven, J. Montgomery, J. A., J. E. Peralta, F. Ogliaro, M. Bearpark, J. J. Heyd, E. Brothers, K. N. Kudin, V. N. Staroverov, R. Kobayashi, J. Normand, K. Raghavachari, A. Rendell, J. C. Burant, S. S. Iyengar, J. Tomasi, M. Cossi, N. Rega, J. M. Millam, M. Klene, J. E. Knox, J. B. Cross, V. Bakken, C. Adamo, J. Jaramillo, R. Gomperts, R. E. Stratmann, O. Yazyev, A. J. Austin, R. Cammi, C. Pomelli, J. W. Ochterski, R. L. Martin, K. Morokuma, V. G. Zakrzewski, G. A. Voth, P. Salvador, J. J. Dannenberg, S. Dapprich, A. D. Daniels, Ö. Farkas, J. B. Foresman, J. V. Ortiz, J. Cioslowski, D. J. Fox, Gaussian, Inc., Wallingford CT, 2009.

S-5. MO distributions and dipole moment in the ground and excited states of ACL and MCP

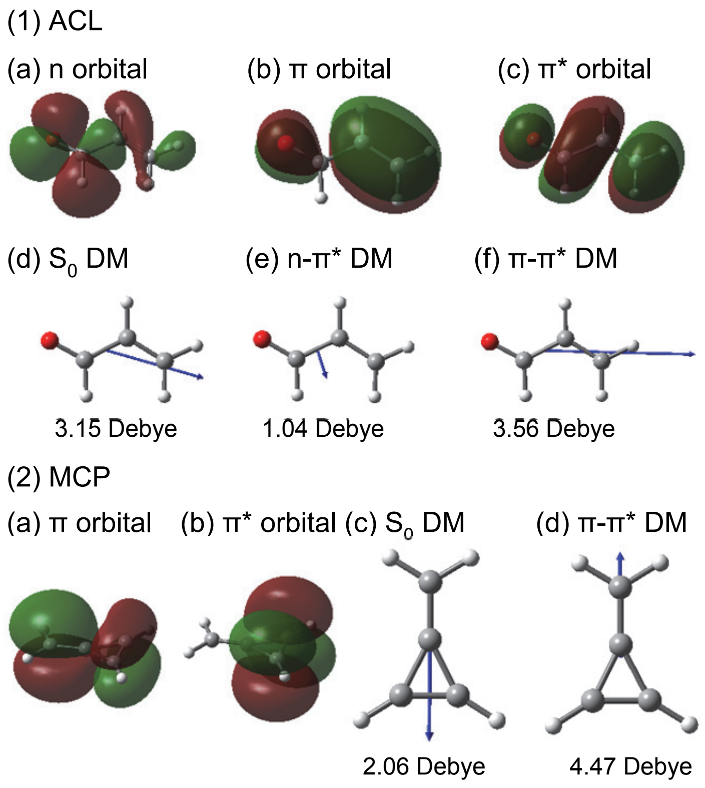

Figure S5. MO distributions and dipole moment in the ground and excited states. (1) (a) n orbital, (b) π orbital, (c) π* orbital, (d) dipole moment of the S_0, (e) S_1, and (f) S_2 states of ACL in gas phase. (2) (a) π orbital, (b) π* orbital, (c) dipole moment in the S_0 and (d) S_1 states of MCP in gas phase. The excited states were calculated at the ground-state energy minimum structure in each molecule. The center of mass was taken as the gauge origin.

S-6. Classical molecular dynamics calculations

Atomic coordinates for ACL and MCP were taken from the snapshot of classical molecular dynamics (MD) calculations. Force field (FF) for ACL and MCP was generated with General Amber FF (GAFF). B3LYP optimized structure and calculated electrostatic potential (ESP) were calculated by Gaussian09 program and used as the input data for the Antechamber program. A periodic boundary condition was used for the solute-in-solvent system. TIP3P model was used. One ACL in 888 H_2O and one MCP in 1164 H_2O were included in a unit cell. With the NPT ensemble, a classical MD was performed at 300 K and 1.0 atm for 5 ns. After equilibration, we took one of the snapshots.

S-7. Tables of the 2nd order contribution to excitation energy

In this section, numerical data of Figures 2 and 3 is tabulated. See Tables S1 and S2 for Figure 2, and Table S3 for Figure 3.

Table S1. The 2nd order contribution of each solvent molecule to the excitation energy for the lowest n-π* of s-trans-acrolein. Unit is eV.

Fragment	Pol+Disp[a]	Polarization	Dispersion
2	-0.003	0.000	-0.002
3	-0.015	-0.004	-0.011
4	-0.005	-0.001	-0.003
5	-0.002	0.000	-0.001
6	-0.002	0.000	-0.001
7	-0.003	0.000	-0.002
8	-0.004	-0.001	-0.003
9	-0.011	-0.003	-0.007
10	-0.002	0.000	-0.002
11	-0.004	-0.001	-0.003
12	-0.005	-0.002	-0.004
13	-0.009	-0.001	-0.008
Total	-0.064	-0.015	-0.049

[a] Total contribution (polarization + dispersion) of each fragment. [b] Total contribution of solvent molecules.

Table S2. The 2nd order contribution of each solvent molecule to the excitation energy for the lowest π-π* of s-trans-acrolein. Unit is eV.

Fragment	Pol+Disp[a]	Polarization	Dispersion
2	-0.005	0.000	-0.005
3	-0.008	-0.001	-0.007
4	-0.003	0.000	-0.003
5	-0.003	0.000	-0.003
6	-0.003	0.000	-0.002
7	-0.004	0.000	-0.004
8	-0.004	0.000	-0.004
9	-0.006	0.000	-0.006
10	-0.002	0.000	-0.001

11	-0.004	0.000	-0.004
12	-0.003	0.000	-0.003
13	-0.011	-0.001	-0.009
Total	-0.054	-0.004	-0.050

[a] Total contribution (polarization + dispersion) of each fragment. [b] Total contribution of solvent molecules.

Table S3. The 2nd order contribution of each solvent molecule to the excitation energy for the lowest *π*-*π state of methylenecyclopropene. Unit is eV.**

Fragment	Pol+Disp[a]	Polarization	Dispersion
2	-0.001	0.000	0.000
3	-0.002	-0.001	-0.001
4	-0.002	-0.001	-0.001
5	-0.009	-0.003	-0.005
6	-0.002	-0.001	-0.001
7	-0.004	-0.003	-0.001
8	-0.026	-0.015	-0.011
9	-0.002	-0.001	-0.001
10	-0.001	-0.001	-0.001
11	-0.002	-0.001	-0.001
12	-0.001	0.000	0.000
13	-0.002	-0.001	-0.002
14	-0.002	-0.001	-0.001
15	-0.001	0.000	-0.001
16	-0.002	-0.001	-0.001
17	-0.004	-0.001	-0.003
18	-0.003	-0.001	-0.002
19	-0.001	0.000	0.000
20	-0.002	-0.001	-0.001
21	-0.003	-0.001	-0.002
22	-0.001	-0.001	-0.001
Total	-0.050	-0.026	-0.025

[a] Total contribution (polarization + dispersion) of each fragment. [b] Total contribution of solvent molecules.

S-8. Atomic coordinates of the computational models

(1) s-trans-acrolein + $12H_2O$

6	0	22.642200	39.870300	26.033300
1	0	21.908500	39.108800	25.745800
1	0	23.613500	39.878200	25.532100
6	0	22.357100	40.757700	26.990300
1	0	23.064900	41.526100	27.308700
6	0	21.065000	40.722000	27.690000
1	0	20.402600	39.857800	27.456700
8	0	20.687600	41.588000	28.460500
8	0	21.936000	40.309000	22.630000
1	0	22.335000	40.892000	21.862000
1	0	21.576000	41.090000	23.141000
8	0	17.886000	40.898000	27.280000
1	0	17.244000	41.565000	27.032000
1	0	18.510000	41.323000	27.880000
8	0	22.250000	44.631000	28.110000
1	0	21.818000	45.443000	28.301000
1	0	21.487000	44.122000	27.631000
8	0	23.670000	38.188000	23.021000
1	0	23.086000	38.955000	22.930000
1	0	23.361000	37.754000	22.178000
8	0	26.032000	41.226000	26.083000
1	0	25.530000	42.043000	26.343000
1	0	26.221000	41.409000	25.158000
8	0	20.068000	38.111000	24.331000
1	0	20.920000	38.404000	24.049000
1	0	19.557000	38.889000	24.278000
8	0	20.323000	44.163000	26.270000
1	0	20.983000	44.001000	25.647000
1	0	19.594000	44.532000	25.692000
8	0	21.355000	42.552000	31.300000
1	0	21.106000	42.569000	30.358000
1	0	21.259000	43.511000	31.497000
8	0	19.071000	37.526000	29.221000
1	0	19.074000	36.608000	29.471000
1	0	18.948000	37.444000	28.249000
8	0	24.249000	42.797000	28.947000
1	0	23.983000	41.878000	28.964000
1	0	23.427000	43.261000	28.829000
8	0	18.418000	43.600000	28.488000
1	0	18.828000	44.125000	27.727000
1	0	19.040000	43.821000	29.201000
8	0	23.306000	40.509000	29.846000
1	0	22.526000	40.419000	30.450000
1	0	23.776000	39.696000	30.175000

(2) methylenecyclopropene + $12H_2O$

6	0	-1.492000	-0.759000	-0.186000
1	0	-2.200000	-0.260000	-0.849000
1	0	-1.769000	-1.722000	0.247000
6	0	-0.292000	-0.215000	0.036000
6	0	0.654000	0.868000	-0.109000
1	0	0.892000	1.840000	-0.537000
6	0	0.987000	-0.124000	0.701000

1	0	1.698000	-0.499000	1.435000
8	0	3.034000	4.771000	2.204000
1	0	2.628000	5.409000	2.774000
1	0	3.764000	4.390000	2.806000
8	0	1.649000	-3.796000	-2.009000
1	0	1.137000	-3.532000	-1.263000
1	0	1.902000	-4.727000	-1.630000
8	0	-3.835000	-4.116000	-2.605000
1	0	-3.761000	-3.353000	-1.981000
1	0	-3.720000	-3.678000	-3.475000
8	0	-0.999000	2.043000	-2.785000
1	0	-0.758000	1.386000	-2.110000
1	0	-0.284000	2.716000	-2.555000
8	0	-4.125000	2.502000	0.283000
1	0	-3.786000	1.995000	1.091000
1	0	-5.060000	2.674000	0.581000
8	0	-4.590000	-1.208000	-1.838000
1	0	-4.003000	-0.466000	-2.038000
1	0	-5.191000	-1.185000	-2.585000
8	0	-2.515000	0.990000	2.347000
1	0	-1.725000	1.363000	2.754000
1	0	-2.127000	0.391000	1.689000
8	0	2.950000	4.245000	-0.487000
1	0	3.069000	4.026000	0.450000
1	0	2.584000	5.174000	-0.385000
8	0	-0.248000	-5.062000	0.674000
1	0	0.617000	-4.766000	0.986000
1	0	-0.831000	-4.728000	1.343000
8	0	1.853000	-3.926000	1.777000
1	0	2.502000	-4.360000	2.410000
1	0	1.472000	-3.191000	2.286000
8	0	4.025000	-2.321000	-2.857000
1	0	4.568000	-2.755000	-2.172000
1	0	3.241000	-2.890000	-2.867000
8	0	3.761000	0.391000	-2.889000
1	0	3.138000	-0.165000	-3.443000
1	0	4.176000	-0.310000	-2.357000
8	0	-3.783000	1.531000	-2.418000
1	0	-4.028000	1.913000	-1.530000
1	0	-2.982000	2.082000	-2.596000
8	0	6.124000	-0.136000	-1.060000
1	0	6.911000	0.288000	-0.696000
1	0	5.410000	0.313000	-0.518000
8	0	4.776000	-1.697000	1.601000
1	0	4.884000	-0.765000	1.315000
1	0	4.985000	-2.192000	0.791000
8	0	-0.252000	2.467000	3.376000
1	0	-0.884000	3.086000	2.889000
1	0	0.239000	3.134000	3.927000
8	0	2.875000	-1.371000	3.660000
1	0	3.810000	-1.015000	3.862000
1	0	3.104000	-1.720000	2.765000
8	0	-2.724000	4.394000	-0.959000
1	0	-2.483000	4.834000	-0.172000
1	0	-3.386000	3.725000	-0.639000
8	0	-4.939000	-0.524000	2.359000
1	0	-4.126000	-0.030000	2.643000
1	0	-4.592000	-1.391000	2.204000
8	0	0.552000	3.841000	-1.867000

1	0	0.402000	4.540000	-1.202000
1	0	1.537000	3.780000	-1.827000
8	0	-3.952000	-3.114000	2.406000
1	0	-2.994000	-3.432000	2.478000
1	0	-4.226000	-3.795000	1.743000

References

[1] J. Hasegawa, T. Kawatsu, K. Toyota, K. Matsuda, *Chem Phys Lett* 2011, *508*, 171-176.
